# Product Factors Affecting Milk Choices among Chinese Older Adults

**DOI:** 10.3390/foods13030371

**Published:** 2024-01-23

**Authors:** Ao Chen, Saleh Moradi, Joanne Hort

**Affiliations:** 1 Food Experience and Sensory Testing (Feast) Laboratory, Massey University, Palmerston North 4442, New Zealand; a.chen2@massey.ac.nz (A.C.);; 2 Riddet Institute, Massey University, Palmerston North 4442, New Zealand; 3 Fonterra Research and Development Centre, Fonterra Co-Operative Group Limited, Palmerston North 4410, New Zealand

**Keywords:** milk targeting older adults, Chinese milk consumption, choice simulation, gifting behaviour

## Abstract

In China, milk is promoted both as an optimal food and gift for older adults. To understand the product factors affecting older Chinese adult milk choices, choice simulations and surveys were conducted in Beijing, Shanghai, Guangzhou, Chengdu, and Shenyang, China. Participants (*n* = 1000, aged 45–55 years old and 65–75 years old) were asked to choose one milk product out of eight alternatives for self-consumption and gifting, respectively, and to indicate product factors under their considerations. Results showed that, for self-consumption, the top four most popular milk products (two with domestic brands and two with international brands) were chosen by 84.9% of the participants. Females and younger participants were more open to international brands than their counterparts. Popular milk products differed across cities, potentially due to brand familiarity. Brand (85.9%), on-the-pack, nutrition-related well-being messaging (72.9%), price (63.1%), shelf-life (63.0%), and production date (57.6%) were the most frequently reported product factors considered when choosing milk. More males considered price than females (66.9% vs. 60.0%, *p* = 0.02). Female and older participants showed greater concern for certain detailed product factors, such as production date and shelf-life, than their counterparts. Variation across cities was limited, with participants in Chengdu and Shenyang showing less concern for certain product factors such as on-the-pack, certificate-related well-being messaging. When milk products were chosen as a gift, although overall milk choice ranking remained similar, package style received increased attention (32.0% vs. 40.8%, *p* < 0.01), whilst all other product factors, especially price (63.1% vs. 49.5%, *p* < 0.01), were considered by significantly fewer participants. These findings provide valuable marketing insights, helping to understand consumer preferences and considerations in the process of milk purchase decision-making.

## 1. Introduction

China used to have one of the lowest levels of milk consumption in the world, at 0.4 kg per capita per year in 1949, and 1.0 kg per capita per year in 1978 [1] (pp. 4–5). However, with the development of the Chinese economy and the influence of Western foods and culture, the Chinese dairy industry has developed rapidly over the last two decades. As of 2022, Chinese milk consumption has reached 42.0 kg milk equivalent per capita per year [2]. Considering its superior nutritional value, particularly in calcium and protein [3], milk has become one of the most popular food products designed for Chinese older adults (COA) [4,5]. In 2021, there were more than 200 milk products targeting COA available in the market, representing 117 manufacturers, both domestic and overseas [6].

In such a competitive market, the key to success depends on understanding consumer preferences and considerations in the process of purchase decision-making. Consumers make their purchase decisions based on the product, situation, context, and previous experiences [7]. Therefore, product factors related to quality in either a concept, content, or context perspective [8] play a critical role in attribute-based choice [9] (pp. 553–555). Previous studies confirmed that product attributes, such as nutrition and packaging, were evaluated by consumers when purchasing [10,11,12]. Additionally, researchers have focused on consumer willingness to pay for various milk product attributes in contingent valuations, such as traceability [13], sustainability [14], organic status [15], and country of origin [16]. Additionally, choice-based conjoint analyses have been conducted on milk products regarding specific product attributes [17], food safety features [18], and brands [19]. However, due to hypothetical bias, responses to hypothetical questions might not approximate actual behaviours [20,21]. Previous studies have shown that the introduction of real economic incentives in choice experiments can mitigate hypothetical bias in terms of behavioural aspects and decision-making processes [22,23,24]. Therefore, in this study, choice simulations with real products and prices were conducted to provide a more realistic and consequential decision-making environment.

Gender and age are often considered as moderators in purchase decision-making. Gender may have an impact on perceptions of quality due to gender role socialisation and differences in information processing [25]. Based on the ‘selectivity model’, females have been identified as comprehensive information processors, who consider both subjective and objective product attributes and respond to subtle cues [26]. In contrast, males are identified as selective information processors, who often use heuristic processing and may overlook subtle cues [26]. Age is also a powerful determinant of consumer behaviour, impacting various aspects, such as interest, sensory preference, purchasing ability, information exposure, learning ability, and influenceability [27,28]. Most importantly, increasing health concern among older adults impacts their dietary choices and preferences compared to younger generations [29,30,31]. Furthermore, societal, economic, and cultural transformations in China’s modern history have fundamentally shaped the life course of different generations and are likely to have influenced their food choice behaviours in later life [32]. Differences in economic development [33,34] and dietary habits across China [35,36] could also potentially influence the food choices of consumers from different regions [37] and their considerations when making purchasing decisions [38]. Significant dependency between the consumption of milk and sociodemographic variables has been established in many countries [39]. For example, in Europe and the US, nationality, ethnicity, and culture appear to influence consumer perception of the importance of food product attributes [40] and have eventually altered milk purchasing decisions [41]. Since milk was not traditionally a part of the Chinese diet, a knowledge gap exists regarding how age, gender, and region of residence may affect COA milk choices. Furthermore, previous studies have investigated the purchase of milk among food categories [42], milk types [41], and brands [43], but seldomly among milk products. This study, however, focuses on choices of specific milk products made by older Chinese consumers across different genders, ages, and cities of residence.

Food symbolises abundance and prosperity in Chinese culture, making it a popular gift option in China [44,45]. Milk’s association with nutrition, health, and family has established it as a regular choice in Chinese gifting culture, especially among older adults [46]. Since Confucianism has deeply shaped Chinese culture and society, the claim that ‘courtesy calls for reciprocity’ (礼尚往来) holds a firm place in Chinese life philosophy [47]. Through giving and exchanging gifts, the Chinese constantly strive to maintain and improve their social networks, i.e., ‘guanxi’ (关系) and ‘face’ (面子), which is the prestige, security, and self-determination enjoyed in social transactions [48]. Therefore, the choices Chinese consumers make when purchasing milk for gifting, as well as their product factor considerations, may differ compared to self-consumption. In a cross-cultural study, Li and Su found that Chinese consumers are more likely to consider the prestige of the products in gift purchasing than for themselves [49]. Qing et al. reported that factors contributing to the self-consumption and gifting of wines in China are different, and sometimes their effects were opposite [50]. For example, more experience with wine led to Chinese consumers choosing less expensive wines for self-consumption but more expensive wines for gifting [50]. However, it is currently unknown whether the product factors considered differ in milk choice for self-consumption versus gifting.

It was hypothesised that: (1) milk choices and the product factors considered when making choices are different among COA consumers of different genders, ages, and cities of residence; and (2) milk choices and the product factors considered are different for self-consumption compared to gifting by COA consumers. Therefore, using choice simulations and surveys, the objectives of this study were (1) to investigate milk choices and the contributing product factors of COA across different genders, ages, and cities; and (2) to identify if the milk choices and product factors considered for gifting purposes are different to self-consumption.

## 2. Materials and Methods

**Scheme 1:**
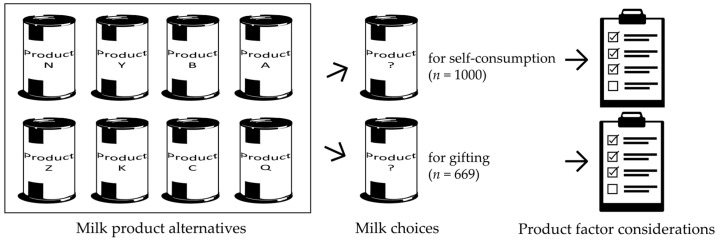
Schematic overview of the research procedure, indicating 8 products presented from which participants selected one (?) for self-consumption, and later one for gifting. On each occasion they indicated via Check-All-That-Apply[☑) which Factors they had considered to make their selection.

The research procedure, outlined in Scheme 1, involved two choice simulations to explore COA milk choices for self-consumption and gifting and subsequent surveys to examine product factors affecting their choices. This study was determined to be low risk by the Massey University Human Ethics Committee (Ethics Notification Number: 4000025653, 11 May 2022). All participants demonstrated informed consent by signing a consent form at the study’s commencement. They were explicitly informed that all data would be anonymised, reported only in aggregate form, and that they were free to withdraw from the study at any time without providing a reason. As compensation for their time, participants were offered remuneration ranging from CNY 70 to CNY 100, depending on their cities of residence. Recruitment, screening, and experiments were conducted by city-based partners of an international consumer and sensory research agency (MMR Management Consulting (Shanghai) Co. Ltd., Shanghai, China) in May and June 2022.

### 2.1. Participants

One thousand regular milk consumer volunteers were recruited from five Chinese cities: Beijing (the capital), Shanghai (east), Guangzhou (south), Chengdu (southwest), and Shenyang (northeast), and across two age groups representing different generations of older adults: 45–55 years old (younger) and 65–75 years old (older). Beijing, Shanghai, and Guangzhou are first-tier cities, whilst Chengdu and Shenyang are second-tier cities. The city tier system is widely used in China, classifying cities primarily based on their level of economic development and population [51]. According to the National Bureau of Statistics [52], the per capita regional gross domestic products of Beijing, Shanghai, and Guangzhou were over twice the national average, whilst Chengdu and Shenyang have per capita regional gross domestic products close to the national average. A preliminary screening survey gathered information on the participant’s weekly milk consumption, gender, age, city residence duration, and general health condition. Eligible participants were regular milk consumers (consuming at least 2 cups or 500 mL of unflavoured or flavoured mammalian milk in either liquid or powdered format weekly), local residents (residing in the city for at least three years), and generally healthy (living independently, not in a nursing home). Quotas were set to ensure an equal number of participants in each of the two age groups across all five cities (*n* = 100). Within each quota, at least 40% female and male participants were recruited. Additionally, data concerning each participant’s education level, monthly income, and employment status were gathered to provide a comprehensive characterisation of the participant sample.

### 2.2. Products and Product Factors

**Figure 1:**
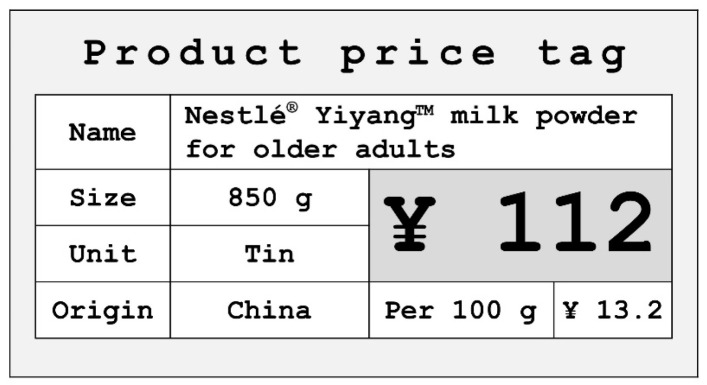
A translated example of a price tag used in choice simulations.

Eight powdered milk products, the dominant milk type for older adults in China [6], were used for the choice simulations. Theoretically, in the purchase decision-making process, consumers gather product information to evaluate possible alternatives based on their knowledge, situation, and previous experiences [9] (pp. 517–580). Under conventional grocery shopping conditions, product information is mainly extracted from price tags and product packages. This information provided the basis to establish a product factor consideration list for the experiment (detailed working definitions with examples are listed in Table 1). Price tags indicated brand, size, unit, product origin, and price (Figure 1), whilst the product packages conveyed mandatory on-the-pack (OTP) well-being messaging (WM), including brand, origin, manufacturer information, nutrition information table, ingredient list, production date, and expiration date, as well as voluntary OTP WM, including nutrition-, health-, ingredient-, brand-, sensory- and certificate-related information [6]. As shown in Table 1, in this study, OTP WM refers to the voluntary information, except that OTP nutrition- and ingredient-related WM also included the mandatory nutrition information table and mandatory ingredient list, respectively.

Milk product alternatives were combinations of the product factors (Table 2), covering a range of brands, prices, origins, sizes, and OTP WM. Product N was selected as a popular international brand [53] produced domestically in China. Product Y and Product B were selected to represent a national domestic brand (Yili) and a regional domestic brand (Bright) local to Eastern China. Notably, Product B portrayed the most OTP WM aspects amongst the eight alternatives. Product A and Product K were selected as international brands from New Zealand, the country from which China imports the most dairy products [1] (p. 21). Despite being imported, Product A was the lowest in price. According to a previous study [6], milk powders targeting COA are also made from other milk sources, particularly goat milk. Consequently, Product Z and Product C were selected, as they were made from goat milk by a domestic and an international company, respectively. Their prices were greater than the cow milk products. Notably, no voluntary WM was presented on the pack of Product C. Finally, a camel milk product, Product Q, was selected to represent minor dairy animals. It was small in package size, but expensive in price. Although different cities received different product batches for the studies, their production dates were all within five months of purchase, as they were obtained from official stores via JD.com (http://global.jd.com, accessed on 12 December 2023), one of the largest Chinese online shopping sites. Product images are provided in Supplementary Materials (Figure S1).

### 2.3. Procedure

All products were positioned with their corresponding price tags in front of the participant. The participant was asked to choose one product for the purpose of self-consumption, as they would normally do during their routine grocery shopping, without an opt-out option, hence forcing an answer. Participants were able to examine the products in detail by picking them up. No additional information about the product was given by the researcher present during the selection. The researcher recorded the choice and subsequently conducted a survey, asking the participant to indicate which product factors were considered based on the product-factor-consideration list shown in Table 1 (in check-all-that-apply format). Subsequently, this process was repeated for the purpose of gifting with participants who had indicated they gifted milk or had thought about gifting milk. The order of products on the table was randomised across participants to minimise order bias. A schematic overview of the research procedure was shown in Scheme 1.

### 2.4. Data Analysis

SPSS Statistics Version 28 (IBM, New York, NY, USA) was used for all analyses with an *a*-risk of 0.05.

To assess the impact of demographic characteristics (gender, age, and city) on milk choices and the product factors considered when choosing milk, chi-square tests were employed. These tests compared the observed response frequencies to the expected frequencies under the null hypothesis. For the ‘city’ variable, if the null hypothesis was rejected, post hoc tests were performed using adjusted residuals with a Bonferroni adjustment to pinpoint which cities were significantly different from their expected frequencies.

To identify the impact of the choice scenario (self-consumption vs. gifting) on milk choice and the product factors considered when choosing milk, chi-square tests were conducted for each milk choice and product factor, respectively, comparing self-consumption and gifting.

## 3. Results

Participant demographics are presented in Table 3. Proportionally, the younger participants and participants from Beijing and Shanghai had higher education levels. Male, younger, and first-tier city (i.e., Beijing, Shanghai, and Guangzhou) participants had higher incomes than their counterparts.

### 3.1. Milk Choices

The ranking of milk product choices for self-consumption is shown in Table 4, with the top four products (Product N, Product Y, Product B, and Product A) accounting for 84.9% of the choices. These products were all made from cow milk and were less expensive than the rest. Out of the 1000 participants, 66.9% had gifted or thought of gifting milk (Table 4). Milk choices for gifting shared a similar pattern to those for self-consumption (Table 4). However, in the gifting scenario, the differences in choice proportions between milk products became smaller, with decreased dominance of popular domestic products such as Product B (17.5% vs. 12.6%, *p* < 0.01) and increased interest in the more expensive, lower-ranking products such as Product K (3.7% vs. 5.8%, *p* = 0.04).

### 3.2. Gender, Age, and City Variation in Milk Choices

As shown in Table 5, more males selected Product Y (29.1% vs. 21.6%, *p* < 0.01), and females tended to choose two of the international brands more frequently (Product N, 31.8% vs. 26.7%, *p* = 0.08; and Product A, 14.4% vs. 11.0%, *p* = 0.11). International products (Product N and Product K) were chosen significantly more often by the younger participants, whilst domestic products (Product Y and Product B) were chosen by more older participants. In terms of the variation among cities, the nation-wide brand, Yili, was chosen more frequently by participants in Beijing (33.5%) and Chengdu (37.5%). The brand local to Shanghai and eastern China, Bright, was more likely to be chosen by participants from the cities located in the east and northeast of China, i.e., Shanghai (34.5%) and Shenyang (25.5%). The proportion of participants selecting Product A was particularly high in Guangzhou (36.5%) and Shanghai (13.5%), compared to the second-tier cities, Chengdu (5.5%) and Shenyang (2.0%).

### 3.3. Product Factors Considered

In Table 6, the data indicated that brand (85.9%), OTP nutrition-related WM (72.9%), price (63.1%), shelf-life (63.0%), and production date (57.6%) were the top five product factors considered for self-consumption by the participants (Table 6). Animal (22.1%), size (33.2%), package style (32.0%), and OTP brand- (36.8%), sensory- (27.4%), certificate- (26.4%), and production-related WM (24.8%) were selected less frequently as considerations in their choices, with origin (45.5%), OTP health- (48.2%) and ingredient-related WM (44.8%) in between. When choosing for gifting, the importance of all product factors considerations decreased, except package style (32.0% vs. 40.8%, *p* < 0.01, Table 6). Considerations of price (63.1% vs. 49.5%, *p* < 0.01), production date (57.6% vs. 47.7%, *p* < 0.01), and shelf-life (63.0% vs. 51.1%, *p* < 0.01) dropped dramatically, compared to choosing for self-consumption.

### 3.4. Gender, Age, and City Variation in Product Factors Considered

More males considered price than females (66.9% vs. 60.0%, *p* = 0.02, Table 7). Female participants, on the other hand, cared more about detailed product factors, such as production date (62.2% vs. 52.1%, *p* < 0.01), shelf-life (67.1% vs. 58.1%, *p* < 0.01), OTP nutrition- (76.4% vs. 68.7%, *p* < 0.01), health–(52.3% vs. 43.3%, *p* < 0.01), ingredient- (47.3% vs. 41.7%, *p* = 0.08), and sensory-related WM (29.6% vs. 24.7%, *p* = 0.08). Participants from the older age group were more likely to consider the following product factors than the younger group: animal (25.4% vs. 18.8%, *p* = 0.01, Table 7), production date (66.6% vs. 48.6%, *p* < 0.01), shelf-life (72.0% vs. 54.0%, *p* < 0.01), and OTP health- (51.2% vs. 45.2%, *p* = 0.06), production- (30.0% vs. 19.6%, *p* < 0.01) and brand-related WM (42.4% vs. 31.2%, *p* < 0.01). Regarding variation amongst cities, the consideration of origin (31.0%), animal (11.0%), production date (45.0%), and OTP certificate-related WM (19.5%) was significantly lower in Chengdu. Participants in Shenyang considered OTP health- (38.5%), production- (15.5%), sensory- (18.5%), and certificate-related WM (24.5%) significantly less. Animal source was considered by more participants in Beijing (34.5%) compared to other cities. More participants considered OTP brand- (47.0%) and certificate-related WM (33.0%) in Shanghai, and origin (55.0%) and OTP certificate-related WM (29.5%) in Guangzhou.

## 4. Discussion

This study, combining choice simulations and relevant survey questions, revealed the ranking of popular milk products by COA and the product factor considerations for either self-consumption or gifting. There were substantial variations in milk choices and product factor considerations amongst participants across different genders, ages and cities of residence, as well as for self-consumption versus gifting purposes.

### 4.1. Milk Choice for Self-Consumption

Amongst the top four most frequently chosen products, Product N and Product A were international brands. Currently, about 15% of milk product varieties targeting COA are produced by international brands in the Chinese market [6]. This finding indicates that certain international brands were as popular a choice as domestic ones.

About 10% of participants chose goat milk (Product Z and Product C combined) and about 2% chose camel milk (Product Q) for self-consumption. Even though about 97.5% of Chinese milk production is from dairy cows [54], alternative milk source consumption has become more popular among the elderly for nourishment and health benefits [55]. In 2022, 41% of the milk product varieties targeting COA in the Chinese market were made from alternative milk sources [6]. The lower ranking of alternative milk sources observed in this study may be caused by their significant price premium [10].

### 4.2. Gender, Age, and City Effects on Milk Choice for Self-Consumption

Participants in the older group, who were most likely influenced by brand familiarity and loyalty patterns [56,57], exhibited a clear preference for domestic products like Product Y and Product B. In contrast, the younger participants showed a higher inclination towards international products, possibly attributed to their broader exposure to diverse products and brands. These findings could be due to older individuals’ limited information exposure and learning ability [28]. Additionally, females tended to choose products with specific international brands as well. These findings align with established patterns of the younger, more educated, higher income individuals, and females being more accepting of imported products [58,59,60]. Gender and age differences in milk choice might also be linked to increased food neophobia in males and older participants, which could stem from limited exposure to a variety of foods during the formation of their dietary habits [61].

Differences in income levels between first- and second-tier city participants (Table 1) did not lead to a preference for more expensive milk in first-tier cities, indicating the relative price inelasticity of milk products (mean price elasticity = 0.95) [62]. It is clear that milk choices were influenced by factors other than price, such as brand preference [63]. Brand familiarity emerged as a key mechanism affecting milk choices amongst COA across cities. For instance, participants from Shanghai and Shenyang were more likely to choose Product B, a brand originating locally or close to their regions. Ding and Veeman’s study also found that only consumers from Chengdu were willing to pay a premium for a brand local to that region [64]. Product A’s higher choice proportion in Guangzhou and Shanghai was attributed to its company (Fonterra’s) strategic response to increasing demand in China, establishing its first two application centres in these cities in 2014 and 2015. Despite not being the most popular in most cities, Product N had the highest overall choice proportion, reflecting Nestlé’s strong brand recognition in China generally [53]. These findings affirm that consumers prefer and trust familiar brands [65]. Theoretically, brand familiarity impacts product choice by influencing cognitive resources and creating conflict when the brand is less familiar [66]. Consumers familiar with a brand can easily relate their needs to its characteristics, whilst unfamiliarity poses difficulties that diminish as knowledge grows [67]. Therefore, brand familiarity plays a significant role in purchase decision-making processes, especially amongst COA with generally lower education levels than younger generations [68].

### 4.3. Product Factors Considered for Self-Consumption

Unsurprisingly, brand was the most considered product factor, which agrees with previous studies [69,70,71]. Brand serves as a sign of quality and helps effectively differentiate products with similar physical characteristics [72,73]. In this study, the variation in milk choices, including between genders and ages and across cities, could all be associated with brand familiarity to some extent. However, Jin et al. reported that only 46.6% of Chinese milk consumers were classified as ‘brand sensitive’ after interviewing adults, including the elderly, across 10 cities in China [38]. Due to the discrepancy between intentions and actions [74], the stated preferences might not entirely align with actual consumer behaviours [20,21]. Results from this study, involving real products and prices, suggest that brand was frequently considered in the purchase decision-making processes of COA when evaluating a range of milk products.

OTP nutrition-related WM was also frequently considered in milk choice, which is in line with findings amongst the Chinese general public. Xu et al. found that nutrition claims and fat content were the top two pieces of product information affecting Chinese consumer preference and willingness to pay for milk products [16]. Jin et al. reported that nutrition was one of the top three product attributes driving consumer milk preferences in Chinese provincial capital cities [38]. The focus on the nutritional profile of milk products also mirrors the primary reason for COA milk consumption, which is centred around nutrition and health [75].

Furthermore, most participants considered shelf-life and production date when choosing milk, even though concerns about food expiration are typically more pronounced for refrigerated products than ambient ones [76]. This attention to shelf-life and production date, especially to the longer shelf-life of powdered milk in this study, could stem from the importance of assessing the quality and safety of fresh milk products [38,77].

In this study, participants paid less attention to the milk’s animal source, which is potentially due to the prevalence of cow milk in the Chinese market [54]. Supporting this explanation, our results showed that only 19.2% of participants (170 out of 886) considered the animal source when choosing cow milk products, whilst this figure more than doubled to 44.7%, if their choice was non-cow milk (51 out of 114), indicating more weight was given to animal source if a consumer specifically wanted an alternative milk source.

The product’s packaging style was one of the least important factors, compared with other product attributes, driving milk choice. Similar results were found for package colour, shape, and transparency, which were significantly less important considerations in milk purchases for self-consumption [38,70].

Notably, certification was determined to be one of the least important considerations among COA. In contrast, organic certification was a discriminatory factor in milk purchasing among young milk consumers with a higher level of education and income in Italy [78]. Other quality certifications were also important considerations in milk purchasing by Italian consumers [71]. Similarly, in a Canadian study, organic certification motivated female consumers (19 to 50 years old) to buy and consume milk [79]. Despite the potential negative impact of age on certification knowledge and awareness [13], the findings in this study were still surprising, especially considering the heightened concerns about food safety and quality in China after the 2008 melamine contamination scandal [80]. The low importance of certification in COA’s milk choices might be due to their unfamiliarity or unawareness, and the lack of credibility of such certificates in the Chinese food market [13]. Consistent with this, Thøgersen et al. reported weaker effects of organic labelling in China compared to other countries [81], and Chen et al. found that certification was not valued by Chinese consumers shopping online for milk targeting older adults [10]. Chinese consumer concern regarding food safety and quality appeared to be reflected more in their trust of specific brands rather than in reliance on certification [64].

Regarding origin, since the melamine contamination scandal [80], studies have consistently highlighted the positive effects of foreign country of origin in Chinese dairy markets due to positive images of the source country as well as the product [16,82]. A choice-based conjoint experiment further confirmed a preference for food products from developed countries over developing countries, particularly within emerging economies, including China [81]. However, in contrast to the aforementioned research, the results of the current study showed that product origin was considered by less than half of COA participants when evaluating and choosing milk products. It seems that the preference for specific overseas products was associated with specific brands rather than their origin. This was further supported by the results of their milk choices, as not all products from the same overseas country of origin were equally popular (Anlene 12.9% vs. Karivita 3.7%, both from New Zealand).

### 4.4. Gender, Age, and City Effects on Product Factors Considered for Self-Consumption

In this study, more males considered price, whilst more females cared about other product details, such as production date, shelf-life, and OTP nutrition-, health-, ingredient-, and brand-related WM. These findings align with research showing that males were proportionally more sensitive to price when shopping for milk [16], making males more susceptible to price manipulation than females [83]. In contrast, females tend to be ‘nutrition claims seekers’ [16,84], and female older adults normally spend more time on food shopping than males [85].

Participants in the older group generally considered more product factors than their younger counterparts. It has been reported that older consumers are generally more thoughtful shoppers and tend to take their time while shopping [25]. However, since information searching by older consumers is less intense and enduring, they invest less time in information searching in specific aspetcs (e.g., nutrition-related), leading to smaller consideration sets [86,87]. Consequently, the longer shopping times and smaller consideration sets resulted in more product factors considered by the older participants than by the younger ones. Therefore, OTP WM covering a wider range of aspects would better fulfil the information-gathering needs of older consumers during the evaluation of alternatives.

In this study, limited heterogeneity was observed in product factor considerations of milk choices across Chinese cities. Given China’s vast territory and regional development variations [88,89], differences in milk consumer preference and behaviour were expected. For example, a prior study identified distinct categories of Chinese consumers in milk choices by comparing participants from provincial capital and non-capital cities [38]. However, the results of this study revealed a similar pattern of product factors considered amongst participants from different cities. This may be attributed to the relatively modest gaps between cities. Greater variations could potentially arise when contrasting consumers in rural and urban China.

### 4.5. Milk Choice for Gifting

Food gifting is common in China as a social exchange [45]. In wine gifting, Chinese consumers almost always prefer international brands to domestic ones [90,91]. In this study, similarly, all international brands were slightly more frequently chosen for gifting than for self-consumption. In addition, there was a slight increase in the choice of goat and camel milk. These findings reflected the increasing interest in more novel and expensive products for gifting than for self-consumption. Gift-givers generally believe in the importance of surprising recipients and demonstrating generosity [92]. Quach and Lee [93] identified four distinct gift-giver segments based on psychographic consumption traits, one of which was novelty seeking, indicating a need to exceed the recipients’ expectations when purchasing gifts [94]. Previous studies also noted that consumers typically spend more on gift purchasing than for self-consumption [50]. An expensive gift communicates positive attitudes from the gift-giver to the recipient and signals a willingness to invest resources in their relationship [95]. Additionally, the tendency of choosing more expensive milk products as gifts in this study also mirrors a Chinese concept that giving away or sacrificing part of one’s resources in ‘guanxi’ exchange results in an increase in one’s ‘face’ or social reputation [48].

### 4.6. Product Factors Considered for Gifting

Choosing milk as a gift reduced the importance of all product factor considerations (particularly price), except for package style, compared to self-consumption. Such findings could be explained by Chinese cultural and societal norms related to gifting, which prioritise certain attributes (such as aesthetics or prestige) over practical considerations [96]. Consequently, food products with packages designed for gifting are prevalent in the Chinese market [97]. Previous studies in China showed that such gift packaging positively influenced consumers’ gift-giving intentions, encouraging gift-giving behaviours by assuring product quality [98].

### 4.7. Limitations and Future Studies

Data indicated significant variations in milk consumption habits across Chinese cities [75]; therefore, substantial effects of city on milk choices and product factor considerations were expected. However, the observed differences were limited, probably due to the narrow range of alternatives, which were all products claiming suitability for older adults. Additionally, as COA have uniquely introduced milk as a food later in life, the findings in this study may not apply to consumers in other countries and age groups.

Further studies could benefit from incorporating eye-tracking technology to pinpoint packaging elements that capture consumer attention. Integrating the results with this study could yield valuable insights into optimal information placement. For example, eye-tracking research showed that the product name, brand, and images on food packages attracted more attention than other features, such as nutrition claims [99,100]. This may explain the inclusion of nutrition claims in product names occasionally, e.g., Product Y and Product B.

## 5. Conclusions

Among the eight alternatives, the top four most popular milk products were chosen by most of the participants. Female and younger COA were more open to the international brands than their counterparts. The popular milk products varied across different cities, potentially associated with brand familiarity. Brand, OTP nutrition-related WM, price, shelf-life, and production date were the most frequently considered product factors by particpants when choosing milk products. The animal, size, package style, and OTP brand-, sensory-, certificate-, and production-related WM were considered less frequently. Gender, age, and city of residence were also identified as factors affecting product factor considerations in milk choice. In the gifting scenario, the overall milk choice ranking remained similar to that of self-consumption, with an increase in the choice of more expensive products from international brands and/or made from alternative animal milks. When choosing milk products for gifting, package style received heightened attention, whilst all the other product factors, especially price, were considered by fewer participants.

There are implications for both domestic and overseas producers interested in the Chinese milk market. First, international brands were as favoured as, if not more popular than, domestic ones. Second, brand was the most critical factor in COA choice of milk products, and variations in milk choices across different genders, ages, and cities could be linked to brand familiarity to varying degrees. Consumers of different genders, ages, and cities prioritised product factors differently, which could call for different strategies in marketing and product development. Finally, the observed changes in milk choices and product factors considered for gifting indicate opportunities for premium products presented in well-designed gifting packages.

## Figures and Tables

**Table 1:** Working definitions and examples from the product factor consideration list.

Product Factor	Definition	Example
Brand	a company name, or the name it invents for a particular series of products	Nestlé; Bright
Price	cost of the product	CNY 112; CNY 98; CNY 12 per 100 g
Origin	place the product was produced	New Zealand; China
Animal	animal from which the product is made	cow; goat
Production date	date on which the product was first produced	22 May 2022; 19 March 2022
Shelf-life	length of time the product remains fit for consumption	360 days; 720 days
Size	net weight of the product	850 g; 900 g
Package style	format and appearance of the product package	box; tin; gift packaging
On-the-pack well-being messaging		
Nutrition-related	information about the product’s nutritional profile and features	nutrition information table; high in calcium; low in fat
Health-related	information about potential health benefits	calcium is good for your bones and teeth; a schematic image of bowel movement
Ingredient-related	information about the source, quality, and characteristics of the ingredients	ingredients list; imported probiotics; milk base imported from New Zealand;
Production-related	information about the production process	production process adheres to the applicable national standards
Brand-related	information about the history, reputation, goal, or strength of the brand	choose quality, choose Nestlé; Bright has a long history
Sensory-related	information about the appearance, texture, aroma, mouthfeel, and taste of the product	Rich and flavourful
Certificate-related	approval or document issued by a third party to an organisation compliant with applicable production regulations	SO9001; HACCP; GMP; Halal

**Table 2:** Detailed information of product alternatives in the choice simulations.

	Product Code							
	N	Y	B	A	Z	K	C	Q
Name	Yiyang™ milk powder for older adults	High calcium and low-fat milk powder for older adults	High calcium and multi-vitamin milk powder for older adults	Classic milk powder for older adults	No-sugar and high-calcium goat milk powder for older adults	Milk powder for older adults	Formulated goat milk powder	Camel milk powder for older adults
Brand	Nestlé	Yili	Bright	Anlene	Zhenmu	Karivita	Cabrilac	Qianjue
Price (CNY/100 g)	13.2	8.7	12.3	8.1	16.6	14.3	57.0	109.3
Brand origin	Switzerland	China	China	New Zealand	China	New Zealand	France	China
Product origin	China	China	China	New Zealand	China	New Zealand	France	China
Animal	Cow	Cow	Cow	Cow	Goat	Cow	Goat	Camel
Shelf-life (month)	24	24	24	24	24	24	36	24
Size (g)	850	850	800	850	820	900	400	300
Package style	Tin	Tin	Tin	Tin	Tin	Tin	Tin	Tin
On-the-pack well-being messaging								
Nutrition-related ^1^	**Present** ^2^	**Present**	**Present**	**Present**	**Present**			**Present**
Health-related	Present			Present	Present			
Ingredient-related ^3^	**Present**	**Present**	**Present**	**Present**	**Present**	Present		**Present**
Production-related			Present					
Brand-related	Present	Present	Present					
Sensory-related			Present					
Certificate-related		Present	Present			**Present**		**Present**

^1^ Excludes mandatory nutrition information table; ^2^ bold means well-being messaging presented on front-of-pack; ^3^ excludes mandatory ingredients list.

**Table 3:** Number and demographic characteristics of participants.

	Gender		Age		City				
	Female	Male	45–55	65–75	Beijing	Shanghai	Guangzhou	Chengdu	Shenyang
Number of participants	547	453	500	500	200	200	200	200	200
Average age	59.4	57.7	49.5	67.7	58.5	58.4	58.6	59.4	58.1
Education									
Primary school and below	4.0%	2.7%	0.8%	6.0%	0.5%	0.5%	9.0%	6.5%	0.5%
Middle school	19.8%	15.7%	2.2%	33.6%	10.5%	8.0%	26.0%	23.0%	22.0%
High school	29.4%	28.0%	14.0%	43.6%	30.0%	28.0%	22.5%	27.5%	36.0%
University and above	46.8%	53.6%	83.0%	16.8%	59.0%	63.5%	42.5%	43.0%	41.5%
Employment									
Working	31.3%	53.9%	82.0%	1.0%	39.0%	41.5%	39.5%	40.0%	47.5%
Retired	68.7%	46.1%	18.0%	99.0%	61.0%	58.5%	60.5%	60.0%	52.5%
Monthly income ^1^									
<CNY 2500	1.6%	0.2%	0.2%	1.8%	0.0%	0.0%	0.5%	1.5%	3.0%
≤CNY 2500 and <CNY 5000	50.3%	28.0%	23.8%	56.6%	31.0%	31.0%	32.5%	46.0%	60.5%
≤CNY 5000 and <CNY 8000	27.6%	31.2%	29.4%	29.0%	28.0%	29.0%	33.5%	30.5%	25.0%
≥CNY 8000	20.5%	40.4%	46.4%	12.6%	40.5%	40.0%	33.5%	22.0%	11.5%
Prefer not to say	0.0%	0.2%	0.2%	0.0%	0.5%	0.0%	0.0%	0.0%	0.0%

^1^ CNY 1 = US $0.15 in May and June 2022.

**Table 4:** Numbers, proportions, and associated chi-square *p*-values of Chinese older adults’ milk choices for self-consumption and gifting.

Product	Self-Consumption (*n* = 1000)		Gifting (*n* = 669)		*p*-Value
	Number	Proportion	Number	Proportion	
N	295	29.5%	206	30.8%	0.63
Y	250	25.0%	146	21.8%	0.16
B	175	17.5%	84	12.6%	<0.01
A	129	12.9%	90	13.4%	0.74
Z	70	7.0%	58	8.7%	0.21
K	37	3.7%	39	5.8%	0.04
C	26	2.6%	26	3.9%	0.14
Q	18	1.8%	20	3.0%	0.11

**Table 5:** Proportions and associated chi-square *p*-value of Chinese older adults’ milk choices across different genders, ages, and cities of residence.

Product	Gender			Age			City					
	Female	Male	*p*-Value	45–55	65–75	*p*-Value	Beijing	Shanghai	Guangzhou	Chengdu	Shenyang	*p*-Value
N	31.8%	26.7%	0.08	36.4%	22.6%	<0.01	28.5%	32.5%	26.5%	33.0%	27.0%	0.46
Y	21.6%	29.1%	<0.01	20.6%	29.4%	<0.01	33.5% ^↑^	9.5% ^↓^	13.5% ^↓^	37.5% ^↑^	31.0%	<0.01
B	16.6%	18.5%	0.43	13.4%	21.6%	<0.01	10.0% ^↓^	34.5% ^↑^	6.0% ^↓^	11.5%	25.5% ^↑^	<0.01
A	14.4%	11.0%	0.11	13.4%	12.4%	0.64	7.0%	13.5%	36.5% ^↑^	5.5% ^↓^	2.0% ^↓^	<0.01
Z	7.9%	6.0%	0.24	6.0%	8.0%	0.22	8.5%	4.0%	8.5%	6.0%	8.0%	0.94
K	3.7%	3.8%	0.94	6.0%	1.4%	<0.01	3.5%	3.5%	4.5%	3.0%	4.0%	0.31
C	2.2%	3.1%	0.38	2.4%	2.8%	0.69	6.0% ^↑^	2.0%	1.5%	2.5%	1.0%	0.02
Q	1.8%	1.8%	0.94	1.8%	1.8%	1.00	3.0%	0.5%	3.0%	1.0%	1.5%	0.20

^↑^, ^↓^ Proportion higher or lower than expected, with Bonferroni correction *α* = 0.05.

**Table 6:** Numbers, proportions, and associated chi-square *p*-values of Chinese older adults’ product factor considerations for self-consumption and gifting.

Product Factor	Self-Consumption (*n* = 1000)		Gifting (*n* = 669)		*p*-Value
	Number	Proportion	Number	Proportion	
Brand	859	85.9%	532	79.5%	<0.01
Price	631	63.1%	331	49.5%	<0.01
Origin	455	45.5%	267	39.9%	0.02
Animal	221	22.1%	111	16.6%	<0.01
Production date	576	57.6%	319	47.7%	<0.01
Shelf-life	630	63.0%	342	51.1%	<0.01
Size	332	33.2%	157	23.5%	<0.01
Package style	320	32.0%	273	40.8%	<0.01
On-the-pack well-being messaging					
Nutrition-related	729	72.9%	482	72.0%	0.70
Health-related	482	48.2%	300	44.8%	0.18
Ingredient-related	448	44.8%	245	36.6%	<0.01
Production-related	248	24.8%	126	18.8%	<0.01
Brand-related	368	36.8%	215	32.1%	0.05
Sensory-related	274	27.4%	170	25.4%	0.37
Certificate-related	264	26.4%	169	25.3%	0.60

**Table 7:** Proportions and associated chi-square *p*-values of Chinese older adults’ product factor considerations for self-consumption across different genders, ages and residing cities.

Product Factor	Gender			Age			City					
	Female	Male	*p*-Value	45–55	65–75	*p*-Value	Beijing	Shanghai	Guangzhou	Chengdu	Shenyang	*p*-Value
Brand	84.3%	87.9%	0.11	86.8%	85.0%	0.41	83.5%	89.0%	81.0%	90.5%	85.5%	0.05
Price	60.0%	66.9%	0.02	63.4%	62.8%	0.84	60.0%	57.0%	69.5%	61.5%	67.5%	0.05
Origin	45.9%	45.0%	0.79	45.2%	45.8%	0.85	46.5%	52.5%	55.0% ^↑^	31.0% ^↓^	42.5%	<0.01
Animal	21.9%	22.3%	0.89	18.8%	25.4%	0.01	34.5% ^↑^	24.5%	20.5%	11.0% ^↓^	20.0%	<0.01
Product date	62.2%	52.1%	<0.01	48.6%	66.6%	<0.01	64.0%	63.0%	61.0%	45.0% ^↓^	55.0%	<0.01
Shelf-life	67.1%	58.1%	<0.01	54.0%	72.0%	<0.01	67.5%	66.5%	63.5%	63.0%	54.5%	0.06
Size	33.6%	32.7%	0.75	34.4%	32.0%	0.42	39.0%	33.5%	31.5%	34.0%	28.0%	0.22
Package style	30.5%	33.8%	0.27	33.0%	31.0%	0.50	30.5%	36.5%	29.0%	35.0%	29.0%	0.34
On-the-pack well-being messaging												
Nutrition-related	76.4%	68.7%	<0.01	75.2%	70.6%	0.10	70.0%	80.5%	73.0%	71.5%	69.5%	0.09
Health-related	52.3%	43.3%	<0.01	45.2%	51.2%	0.06	44.5%	56.0%	54.5%	47.5%	38.5% ^↓^	<0.01
Ingredient-related	47.3%	41.7%	0.08	42.6%	47.0%	0.16	44.0%	52.5%	39.5%	43.5%	44.5%	0.12
Production-related	25.2%	24.3%	0.73	19.6%	30.0%	<0.01	32.0%	30.5%	20.0%	26.0%	15.5% ^↓^	<0.01
Brand-related	37.8%	35.5%	0.45	31.2%	42.4%	<0.01	39.5%	47.0% ^↑^	34.0%	33.5%	30.0%	<0.01
Sensory-related	29.6%	24.7%	0.08	25.6%	29.2%	0.20	28.0%	33.0%	27.0%	30.5%	18.5% ^↓^	0.02
Certificate-related	26.1%	26.7%	0.84	22.8%	30.0%	0.10	25.5%	33.0% ^↑^	29.5% ^↑^	19.5% ^↓^	24.5% ^↓^	0.03

^↑^, ^↓^ Proportion higher or lower than expected, with Bonferroni correction *α* = 0.05.

## Data Availability

Data is contained within the article and supplementary materials.

## Supplementary Materials

The following supporting information can be downloaded at: https://www.mdpi.com/article/10.3390/foods13030371/s1, Figure S1: Front-of-pack images of product alternatives in the choice simulations.

## References

[1] Liu L., Chen B., Dairy Association of China (2019). China Dairy Industry.

[2] Liu Y., Wang J., Dairy Association of China (2023). China Dairy Industry Quality Report.

[3] Wiley A. (2015). Re-Imagining Milk: Cultural and Biological Perspectives.

[4] Wu J., Hao J., Ma Y., Li S. (2020). Elderly Dysphagia and Geriatric Food Development. Farm. Prod. Proc..

[5] Qiang W., Zhang L., Ying X., Li H., Xuan M., Li J. (2020). Development Status and Formula Design of Food for Elders. Sci. Technol. Cereal Oils Foods.

[6] Chen A., Moradi S., Hort J. (2022). On-the-Pack Voluntary Well-Being Messaging for Milks Targeting Chinese Older Adults: A Content Analysis. Foods.

[7] Erasmus A., Boshoff E., Rousseau G. (2001). Consumer Decision-Making Models within the Discipline of Consumer Science: A Critical Approach. J. Fam. Econ. Consum. Sci..

[8] Okpala C.O.R., Korzeniowska M. (2020). Concept, Content, and Context Perspectives of Quality of Agrofood Products: Reflections on Some Consumer Decision-Making-Purchase Scenarios. Front. Nutr..

[9] Hawkins D.I., Mothersbaugh D.L., Gordon B., Hughes D. (2010). Consumer Behaviour: Building Marketing Strategy.

[10] Chen A., Moradi S., Hort J. (2023). Evaluating Front-of-Pack Voluntary Well-Being Messaging for Milk Powders Targeting Chinese Older Adults: A Hedonic Price Model. J. Dairy Sci..

[11] Loke M.K., Xu X., Leung P. (2015). Estimating Organic, Local, and Other Price Premiums in the Hawaii Fluid Milk Market. J. Dairy Sci..

[12] Bimbo F., Bonanno A., Liu X., Viscecchia R. (2016). Hedonic Analysis of the Price of UHT-Treated Milk in Italy. J. Dairy Sci..

[13] Zhang C., Bai J., Wahl T.I. (2012). Consumers’ Willingness to Pay for Traceable Pork, Milk, and Cooking Oil in Nanjing, China. Food Control.

[14] Gao Z., Li C., Bai J., Fu J. (2020). Chinese Consumer Quality Perception and Preference of Sustainable Milk. China Econ. Rev..

[15] Yue F., Arbiol J., Nomura H., Yabe M. (2015). Preferences and Willingness to Pay for Organic Milk among Urban Consumers in Dalian, China. J. Fac. Agric. Kyushu Univ..

[16] Xu L., Yang X., Wu L. (2020). Consumers’ Willingness to Pay for Imported Milk: Based on Shanghai, China. Int. J. Environ. Res. Public Health.

[17] Harwood W.S., Drake M.A. (2020). Validation of Fluid Milk Consumer Segments Using Qualitative Multivariate Analysis. J. Dairy Sci..

[18] Mirosa M., Liu Y., Bremer P. (2020). Determining How Chinese Consumers That Purchase Western Food Products Prioritize Food Safety Cues: A Conjoint Study on Adult Milk Powder. J. Food Prod. Mark..

[19] Velčovská Š., Larsen F.R. (2021). The Impact of Brand on Consumer Preferences of Milk in Online Purchases: Conjoint Analysis Approach. Acta Univ. Agric. Silvi. Mendel. Brun..

[20] Hensher D.A. (2010). Hypothetical Bias, Choice Experiments and Willingness to Pay. Transport. Res. B-Meth..

[21] Meyerhoff J. (2006). Stated Willingness to Pay as Hypothetical Behaviour: Can Attitudes Tell Us More?. J. Environ. Plann. Manag..

[22] Nandonde S.W., Msuya E.E., Mtenga L.A., Kilima F.T., Alphonce R. (2013). The Influence of Incentives in Eliminating Hypothetical Bias: Evidence from a Choice-Based Conjoint Experiment for Beef Products in Iringa and Mbeya Regions in Tanzania. J. Agric. Econ. Dev..

[23] Mørkbak M.R., Olsen S.B., Campbell D. (2014). Behavioral Implications of Providing Real Incentives in Stated Choice Experiments. J. Econ. Psychol..

[24] Fifer S., Rose J., Greaves S. (2014). Hypothetical Bias in Stated Choice Experiments: Is It a Problem? And if so, How Do We Deal with It?. Transport. Res. A-Pol..

[25] Ganesan-Lim C., Russell-Bennett R., Dagger T. (2008). The Impact of Service Contact Type and Demographic Characteristics on Service Quality Perceptions. J. Serv. Mark..

[26] Darley W.K., Smith R.E. (1995). Gender Differences in Information Processing Strategies: An Empirical Test of the Selectivity Model in Advertising Response. J. Advert..

[27] Neal C., Quester P., Hawkins D.I., Hunter C., Partnership B. (2005). Consumer Behaviour: Implications for Marketing Strategy.

[28] Phillips L.W., Sternthal B. (1977). Age Differences in Information Processing: A Perspective on the Aged Consumer. J. Mark. Res..

[29] Liu R., Grunert K.G. (2020). Satisfaction with Food-Related Life and Beliefs about Food Health, Safety, Freshness and Taste among the Elderly in China: A Segmentation Analysis. Food Qual. Prefer..

[30] Falk L.W., Bisogni C.A., Sobal J. (1996). Food Choice Processes of Older Adults: A Qualitative Investigation. J. Nutr. Educ..

[31] Browning C.J., Qiu Z., Yang H., Zhang T., Thomas S.A. (2019). Food, Eating, and Happy Aging: The Perceptions of Older Chinese People. Front. Public Health.

[32] Furst T., Connors M., Bisogni C.A., Sobal J., Falk L.W. (1996). Food Choice: A Conceptual Model of the Process. Appetite.

[33] Yang Z., Xiao X., Cheng G. (2021). The Impact of Urban Household Income on the Consumption Structure of Dairy Products in China. Agric. Technol. Econ..

[34] Cheng R., Wang Q., Wei L. (2022). Income Growth, Employment Structure Transition and the Rise of Modern Markets: The Impact of Urbanization on Residents’ Consumption of Dairy Products in China. PLoS ONE.

[35] Ma P. (2010). The Influence of Geographical Environment upon Dietetic Culture Differences between the North and the South in China. J. Kaili Uni..

[36] Melnick M., Marazita M.L. (1998). Neural Tube Defects, Methylenetetrahydrofolate Reductase Mutation, and North/South Dietary Differences in China. J. Cran. Genet. Dev. Bio..

[37] Fuller F.H., Beghin J.C., Rozelle S. (2007). Consumption of Dairy Products in Urban China: Results from Beijing, Shangai and Guangzhou. Aust. J. Agric. Resour. Econ..

[38] Jin S., Yuan R., Zhang Y., Jin X. (2019). Chinese Consumers’ Preferences for Attributes of Fresh Milk: A Best–Worst Approach. Int. J. Environ. Res. Public Health.

[39] Kurajdova K., Táborecka-Petrovicova J. (2015). Literature Review on Factors Influencing Milk Purchase Behaviour. Int. Rev. Manag. Market..

[40] Dekhili S., Sirieix L., Cohen E. (2011). How Consumers Choose Olive Oil: The Importance of Origin Cues. Food Qual. Prefer..

[41] Gulseven O., Wohlgenant M. (2017). What Are the Factors Affecting the Consumers’ Milk Choices?. Agric. Econ..

[42] De Alwis A., Edirisinghe J.C., Athauda A. (2009). Analysis of Factors Affecting Fresh Milk Consumption among the Mid-Country Consumers. Trop. Agric. Res. Ext..

[43] Kurajdová K., Táborecká-Petrovičová J., Kaščáková A. (2015). Factors Influencing Milk Consumption and Purchase Behavior—Evidence from Slovakia. Proc. Econ. Financ..

[44] Lin L. (2017). Food Souvenirs as Gifts: Tourist Perspectives and Their Motivational Basis in Chinese Culture. J. Tour. Cult. Chang..

[45] Balass J. (2017). Successful Gifting Strategies in China: Analysis of the Millennials in the Food Market. Master’s Thesis.

[46] Wilken L., Knudsen A.-C.L. (2008). Milk, Myth and Magic: The Social Construction of Identities, Banalities and Trivialities in Everyday Europe. Kontur.

[47] Seidemann V., Atwal G., Heine K., Capitello R., Charters S., Menival D., Yuan J. (2017). Gift Culture in China: Consequences for the Fine Wine Sector. The Wine Value Chain in China.

[48] Yang M.M.-H. (1989). The Gift Economy and State Power in China. Comp. Stud. Soc. Hist..

[49] Li J.J., Su C. (2007). How Face Influences Consumption—A Comparative Study of American and Chinese Consumers. Int. J. Market Res..

[50] Qing P., Xi A., Hu W. (2015). Self-Consumption, Gfting, and Chinese Wine Consumers. Can. J. Agric. Econ..

[51] Wu L., Bian Y. (2018). Housing, Consumption and Monetary Policy: How Different Are the First-, Second- and Third-Tier Cities in China?. Appl. Econ. Lett..

[52] National Bureau of Statistics GDP. https://data.stats.gov.cn/search.htm?s=GDP.

[53] Allen L.L. (2010). Chocolate Fortunes: The Battle for the Hearts, Minds, and Wallets of China’s Consumers. Thunderbird Int. Bus. Rev..

[54] Nie S., Hu Y., China Animal Husbandry and Veterinary Yearbook Editorial Committee (2021). China Animal Husbandry and Veterinary Yearbook 2021.

[55] Lanfranchi M., Zirilli A., Passantino A., Alibrandi A., Giannetto C. (2017). Assessment of Milk Consumer Preferences: Identifying the Choice Factors through the Use of a Discrete Logistic Model. Brit. Food J..

[56] Monroe K.B. (1976). The Influence of Price Differences and Brand Familiarity on Brand Preferences. J. Consum. Res..

[57] Lambert-Pandraud R., Laurent G. (2010). Why Do Older Consumers Buy Older Brands? The Role of Attachment and Declining Innovativeness. J. Marking.

[58] Bryant B.E., Cha J. (1996). Crossing the Threshold. Market. Res..

[59] Curtis K.R., Mccluskey J.J., Wahl T.I. (2007). Consumer Preferences for Western-Style Convenience Foods in China. China Econ. Rev..

[60] Juric B., Worsley A. (1998). Consumers’ Attitudes towards Imported Food Products. Food Qual. Prefer..

[61] Siegrist M., Hartmann C., Keller C. (2013). Antecedents of Food Neophobia and Its Association with Eating Behavior and Food Choices. Food Qual. Prefer..

[62] Andreyeva T., Long M.W., Brownell K.D. (2010). The Impact of Food Prices on Consumption: A Systematic Review of Research on the Price Elasticity of Demand for Food. Am. J. Public Health.

[63] Sano N., Tamura S., Yada K., Suzuki T. (2014). Evaluation of Price Elasticity and Brand Loyalty in Milk Products. Procedia Comput. Sci..

[64] Ding Y., Veeman M.M. (2019). Chinese Consumers’ Preferences for Quality Signals on Fresh Milk: Brand versus Certification. Agribusiness.

[65] Kwun D.J.-W., Oh H. (2007). Consumers’ Evaluation of Brand Portfolios. Int. J. Hosp. Manag..

[66] Ma Q., Wang M., Da Q. (2021). The Effects of Brand Familiarity and Product Category in Brand Extension: An ERP Study. Neurosci. Res..

[67] Loginova O. (2010). Brand Familiarity and Product Knowledge in Customization. Int. J. Econ. Theory.

[68] Treiman D.J. (2013). Trends in Educational Attainment in China. Chin. Sociol. Rev..

[69] Uzundumlu A.S., Birinci A., Kurtoğlu S. (2018). Analysis of Factors Affecting Consumers in UHT Milk Consumption: The Case Study of Erzurum. Turk. J. Agric. Food Sci. Technol..

[70] Merlino V.M., Brun F., Versino A., Blanc S. (2020). Milk Packaging Innovation: Consumer Perception and Willingness to Pay. AIMS Agric. Food.

[71] Merlino V.M., Massaglia S., Borra D., Mimosi A., Cornale P. (2022). Which Factors Drive Consumer Decisions during Milk Purchase? New Individuals’ Profiles Considering Fresh Pasteurized and UHT Treated Milk. Foods.

[72] Vranešević T., Stančec R. (2003). The Effect of the Brand on Perceived Quality of Food Products. Brit. Food J..

[73] Valjaskova V., Kral P. (2019). The Importance of Brand in Consumer Buying Behavior and Product Quality Assesment. CBU Int. Conf. Proc..

[74] Ajzen I., Brown T.C., Carvajal F. (2004). Explaining the Discrepancy between Intentions and Actions: The Case of Hypothetical Bias in Contingent Valuation. Pers. Soc. Psychol. B..

[75] Chen A., Moradi S., Huang J., Xu S., Sismey M., Hort J. (2023). Chinese Older Adults’ Milk Consumption Habits: A Study across Five Cities. J. Dairy Sci..

[76] Van Boxstael S., Devlieghere F., Berkvens D., Vermeulen A., Uyttendaele M. (2014). Understanding and Attitude Regarding the Shelf Life Labels and Dates on Pre-Packed Food Products by Belgian Consumers. Food Control.

[77] Wang Z., Mao Y., Gale F. (2008). Chinese Consumer Demand for Food Safety Attributes in Milk Products. Food Policy.

[78] Merlino V.M., Borra D., Lazzarino L.L., Blanc S. (2019). Does the Organic Certification Influence the Purchasing Decisions of Milk Consumers?. Qual. Access Success.

[79] Lacroix M.-J., Desroches S., Turcotte M., Painchaud Guérard G., Paquin P., Couture F., Provencher V. (2016). Salient Beliefs among Canadian Adults Regarding Milk and Cheese Consumption: A Qualitative Study Based on the Theory of Planned Behaviour. BMC Nutr..

[80] Li S., Sijtsema S.J., Kornelis M., Liu Y., Li S. (2019). Consumer Confidence in the Safety of Milk and Infant Milk Formula in China. J. Dairy Sci..

[81] Thøgersen J., Pedersen S., Aschemann-Witzel J. (2019). The Impact of Organic Certification and Country of Origin on Consumer Food Choice in Developed and Emerging Economies. Food Qual. Prefer..

[82] Xu X., Comello M.L.G., Lee S., Clancy R. (2020). Exploring Country-of-Origin Perceptions and Ethnocentrism: The Case of U.S. Dairy Marketing in China. J. Food Prod. Mark..

[83] Privitera G.J., Gillespie J.J., Zuraikat F.M. (2018). Impact of Price Elasticity on the Healthfulness of Food Choices by Gender. Health Educ. J..

[84] Cavaliere A., Ricci E.C., Banterle A. (2015). Nutrition and Health Claims: Who Is Interested? An Empirical Analysis of Consumer Preferences in Italy. Food Qual. Prefer..

[85] Cantaragiu R. (2019). The Impact of Gender on Food Waste at the Consumer Level. Stud. Univ. Vasile Goldis Arad-Econ. Ser..

[86] Lambert-Pandraud R., Laurent G., Lapersonne E. (2005). Repeat Purchasing of New Automobiles by Older Consumers: Empirical Evidence and Interpretations. J. Mark..

[87] Hettich D., Hattula S., Bornemann T. (2018). Consumer Decision-Making of Older People: A 45-Year Review. Gerontologist.

[88] Guo R. (2017). Natural and Environmental Constraints. How the Chinese Economy Works.

[89] Démurger S., Sachs J.D., Woo W.T., Bao S., Chang G., Mellinger A. (2002). Geography, Economic Policy, and Regional Development in China. Asian Econ. Pap..

[90] Yu Y., Sun H., Goodman S., Chen S., Ma H. (2009). Chinese Choices: A Survey of Wine Consumers in Beijing. Int. J. Wine Bus. Res..

[91] Liu F., Murphy J. (2007). A Qualitative Study of Chinese Wine Consumption and Purchasing. Int. J. Wine Bus. Res..

[92] Galak J., Givi J., Williams E.F. (2016). Why Certain Gifts Are Great to Give but Not to Get: A Framework for Understanding Errors in Gift Giving. Curr. Dir. Psychol. Sci..

[93] Quach X., Lee S.H. (2021). Profiling Gifters via a Psychographic Segmentation Analysis: Insights for Retailers. Int. J. Retail Distr. Manag..

[94] Khalmetski K., Ockenfels A., Werner P. (2015). Surprising Gifts: Theory and Laboratory Evidence. J. Econ. Theory.

[95] Mayet C., Pine K.J. The Psychology of Gift Exchange. http://karenpine.com/wp-content/uploads/2011/07/The-Psychology-of-Gift-Exchange.pdf.

[96] Wu R., Steffel M., Shavitt S. (2021). Buying Gifts for Multiple Recipients: How Culture Affects Whose Desires Are Prioritized. J. Bus. Res..

[97] Shi Z., Chang Y., Hao Y., Zhang L., Li X., Zhang P., Pan M. (2023). Tapping the Environmental Potential of Gift Packaging: Implications of Mooncake in China. Int. J. Life Cycle Assess..

[98] Yang Y., Paladino A. (2015). The Case of Wine: Understanding Chinese Gift-Giving Behavior. Market. Lett..

[99] Rebollar R., Lidón I., Martín J., Puebla M. (2015). The Identification of Viewing Patterns of Chocolate Snack Packages Using Eye-Tracking Techniques. Food Qual. Prefer..

[100] Varela P., Antúnez L., Silva Cadena R., Giménez A., Ares G. (2014). Attentional Capture and Importance of Package Attributes for Consumers’ Perceived Similarities and Differences among Products: A Case Study with Breakfast Cereal Packages. Food Res. Int..

