# Supplementary material for: Product Factors Affecting Milk Choices among Chinese Older Adults
Source: Foods. 2024 Jan 23;13(3):371. doi: 10.3390/foods13030371 (PMC10855976; doi:10.3390/foods13030371)
Supplement: Supplementary file 1 [file foods-13-00371-s001.zip › foods-2800315-supplementary.pdf]

Supplementary Material

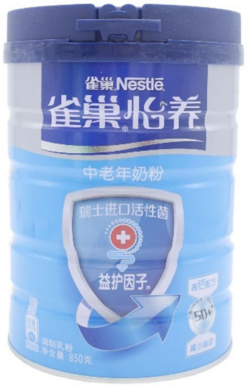

Product N

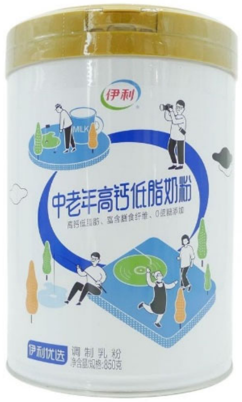

Product Y

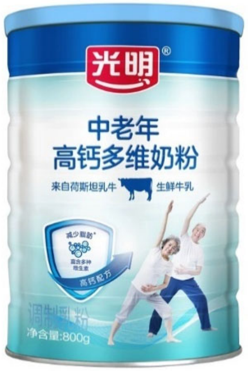

Product B

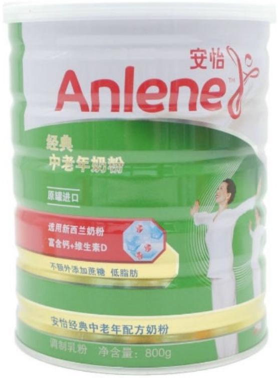

Product A

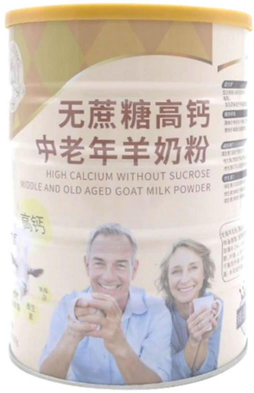

Product Z

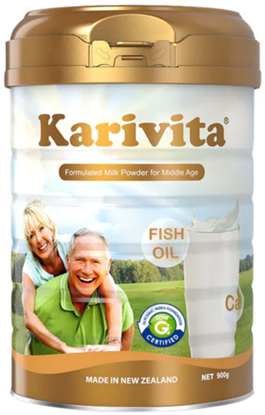

Product K

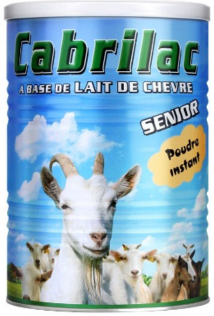

Product C

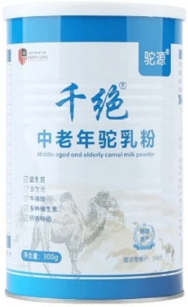

Product Q

Figure S1. Front-of-pack images of the product alternatives in choice simulations. Images have received permissions for use from Mintel Group Ltd.
